# Response of Core Microbial Consortia to Chronic Hydrocarbon Contaminations in Coastal Sediment Habitats

**DOI:** 10.3389/fmicb.2016.01637

**Published:** 2016-10-13

**Authors:** Mathilde Jeanbille, Jérôme Gury, Robert Duran, Jacek Tronczynski, Hélène Agogué, Olfa Ben Saïd, Jean-François Ghiglione, Jean-Christophe Auguet

**Affiliations:** ^1^Equipe Environnement et Microbiologie, Institut Pluridisciplinaire de Recherche sur l’Environnement et les Matériaux, UMR 5254 Centre National de la Recherche Scientifique – Université de Pau et des Pays de L’AdourPau, France; ^2^Laboratoire Biogéochimie des Contaminants Organiques, Unité Biogéochimie et Ecotoxicologie, Département Ressources Biologiques et Environnement, Ifremer Centre AtlantiqueNantes, France; ^3^Littoral, Environnement et Sociétés, UMR 7266 Centre National de la Recherche Scientifique – Université de La RochelleLa Rochelle, France; ^4^Laboratoire de Bio-surveillance de l’Environnement, Faculté des Sciences de BizerteZarzouna, Tunisie; ^5^Laboratoire d’Océanographie Microbienne, Sorbonne Universités, UMR 7621, Centre National de la Recherche Scientifique-University Pierre and Marie CurieBanyuls sur mer, France; ^6^Marine Biodiversity, Exploitation and Conservation, UMR Centre National de la Recherche Scientifique 9190Montpellier, France

**Keywords:** co-occurrence network, core community, microbial consortia, PAH, chronic contamination, coastal sediment

## Abstract

Traditionally, microbial surveys investigating the effect of chronic anthropogenic pressure such as polyaromatic hydrocarbons (PAHs) contaminations consider just the alpha and beta diversity and ignore the interactions among the different taxa forming the microbial community. Here, we investigated the ecological relationships between the three domains of life (i.e., *Bacteria, Archaea*, and *Eukarya*) using 454 pyrosequencing on the 16S rRNA and 18S rRNA genes from chronically impacted and pristine sediments, along the coasts of the Mediterranean Sea (Gulf of Lion, Vermillion coast, Corsica, Bizerte lagoon and Lebanon) and the French Atlantic Ocean (Bay of Biscay and English Channel). Our approach provided a robust ecological framework for the partition of the taxa abundance distribution into 859 core Operational taxonomic units (OTUs) and 6629 satellite OTUs. OTUs forming the core microbial community showed the highest sensitivity to changes in environmental and contaminant variations, with salinity, latitude, temperature, particle size distribution, total organic carbon (TOC) and PAH concentrations as main drivers of community assembly. The core communities were dominated by *Gammaproteobacteria* and *Deltaproteobacteria* for *Bacteria*, by *Thaumarchaeota, Bathyarchaeota* and *Thermoplasmata* for *Archaea* and *Metazoa* and *Dinoflagellata* for *Eukarya*. In order to find associations among microorganisms, we generated a co-occurrence network in which PAHs were found to impact significantly the potential predator – prey relationship in one microbial consortium composed of ciliates and *Actinobacteria*. Comparison of network topological properties between contaminated and non-contaminated samples showed substantial differences in the network structure and indicated a higher vulnerability to environmental perturbations in the contaminated sediments.

## Introduction

Marine sediments cover more than two-thirds of the Earth’s surface. Microorganisms play a central role in global biogeochemical cycling within these habitats (Nealson, 1997; Reed and Martiny, 2013), but also in the biodegradation of undesirable compounds (Wiatrowski and Barkay, 2005; Fernández-Luqueño et al., 2010). Yet, the immense complexity of microbial communities have prevented the precise description of the species involved in these processes and how microbial communities respond to common contaminants such as polyaromatic hydrocarbons (PAHs; Nogales et al., 2011). These questions are particularly relevant in the coastal ecosystems, which support numerous human activities exerting considerable anthropogenic pressures. Hydrocarbon pollution constitutes the most significant of these pressures, as it is estimated to represent worldwide between 1.3 and 8.8 millions tons of discharge per year (Oil in the Sea III, 2003). Estimations of oil discharge in the Mediterranean sea (European Environment Agency, 2006) show that a major part of this pollution is of chronic origin.

The response of microbial communities to chronic inputs of PAHs (Nogales et al., 2011; Sun et al., 2012, 2013; Acosta-González et al., 2013; Sauret et al., 2015) has received little attention compared to the effect of acute contaminations (Head et al., 2006; Stauffert et al., 2013, 2014a,b; Kimes et al., 2014; King et al., 2015). Although no general trend has emerged yet, sediments chronically impacted by PAHs evidenced contrasted variations in bacterial alpha diversity (Nogales et al., 2007; Paissé et al., 2008; Zhang et al., 2008; Païssé et al., 2010; Rosano-Hernández et al., 2012; Sun et al., 2012, 2013), and in community composition, with *Deltaproteobacteria* and *Gammaproteobacteria* as dominant taxa (Paissé et al., 2008; Zhang et al., 2008; Ben Said et al., 2010; Sun et al., 2013). As compared to bacteria, only poor attention is paid nowadays to the response of archaea and eukaryotes to hydrocarbons inputs (Head et al., 2006; Dias et al., 2011; Stauffert et al., 2014b).

All the studies investigating the response of microbial communities to hydrocarbon contaminations have focused mainly on the abundance of taxa found in individual samples (i.e., alpha diversity) or on the turnover of taxa among site (i.e., beta diversity). However, the different species inhabiting any particular habitat interact with each other through flows of matter, energy or information, forming complex ecological networks and these interactions may be more important for ecosystem functioning than richness or abundance (Montoya et al., 2006). Hence, in addition to its alpha and beta diversity facets, biodiversity encompass also the ecological relationships (i.e., direct or indirect interactions) among taxa (Olesen et al., 2007). While plant and macro-organism ecologists have investigated ecological networks for long times (Dunne et al., 2002a,b; Montoya et al., 2006), the recent interest of microbial ecologists for this kind of approach have permitted to ascertain the functional role, the trophic interactions or the ecological niche of uncultured microorganisms (Chaffron et al., 2010; Steele et al., 2011; Barberán et al., 2012; Fillol et al., 2016).

Here we used network analyses in order to explore microbial co-occurrences between the three domains of life in coastal sediments bearing chronic hydrocarbon inputs. We tested the hypothesis of: (i) the existence of core microbial Operational taxonomic units (OTUs) that respond to chronic hydrocarbon contaminations and (ii) an impact of chronic PAH contaminations on the topology of microbial networks. We used 454 pyrosequencing on the 16S rRNA and 18S rRNA genes from coastal sediment microbial communities sampled in chronically impacted and pristine sediments, along the coasts of the Mediterranean Sea and the French Atlantic Ocean.

## Materials and Methods

### Study Sites and Sampling

**FIGURE 1:**
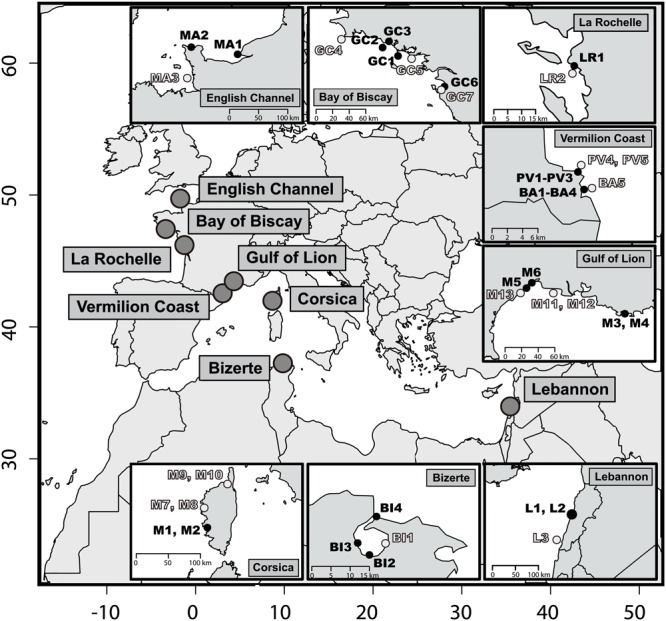
**Map of the 42 sediments sampling stations.** Within each location *a priori* non-contaminated sites are in light gray and *a priori* contaminated sites in black (i.e., contamination criterion defined before sampling). The *x-* and *y-*axes stand for decimal longitude and latitude, respectively.

Forty-two sediment samples were collected in different sites along the Mediterranean and the Atlantic French coasts (**Figure 1**, Supplementary Table S1). An *a priori* contamination criterion was determined according to the vicinity of potential input of hydrocarbons, like urbanized areas, industrial activities, industrial or recreational shipping activities. According to this criterion, contaminated and non-contaminated surface sediment samples (1–2 cm) were collected within each site. Non-contaminated samples were collected at least 2 km away from contaminated sites. Sites from Le Havre (samples MA1 and MA2), Lorient (GC1 and GC2), La Rochelle (LR1), Port-Vendres (PV1 to PV3), Thau lagoon (M5 and M6), Toulon (M3 and M4), Beirut (L1 and L2), Bizerte (BI1) and Ajaccio (M1 and M2) were subjected to industrial activities and industrial shipping (**Figure 1**). Sites from Banyuls-sur-Mer (BA1 et BA4) and Quiberon (GC1) were subjected to recreational shipping activities. Finally, GC6 and GC7 samples were collected in the same location before (August 1999) and after (July 2000) the Erika oil spill, which occurred in December 1999.

Samples were frozen immediately and transported at -18°C to the laboratory, where they were conserved at -80°C until further analysis. Salinity, temperature, particle size distribution (PSD, % < 63 μm), total organic carbon content (%TOC) and PAH concentrations were different among sites (Supplementary Table S1). PSD was measured using laser diffraction, according to Azoury et al. (2013) and %TOC was determined according to Tronczynski et al. (2005), on the decarbonated sediment samples by flash combustion (900–1000°C) with thermal conductivity detection (TCD). PSD was not determined for Bizerte samples, two samples from Lebanon and samples from La Rochelle. %TOC was not determined for two samples from Lebanon, one from the Gulf of Lion, three from Corsica and two samples from La Rochelle (Supplementary Table S1). PAH analyses were processed according to Stauffert et al. (2013) using Gas-Chromatography coupled to Mass-Spectrometry (GC-MS). As PAH concentrations were significantly correlated (rho > 0.75, *p* < 0.05), we were able to calculate PAH sums and compare them with several Sediment Quality Guidelines (SQG, Buchman, 2008): PEL (Probable Effect Low), ERL (Effect Range Low) and T50 (50% toxicity concentration). Two PAH diagnosis ratios [phenanthrene/anthracene (P/A) and fluoranthene/pyrene (F/P)] were considered in order to evaluate the source of contamination across sites. Fluoranthene and phenanthrene are found in fossil fuels and are therefore petrogenic PAHs, while anthracene and pyrene are produced during combustions and are pyrogenic PAHs. Values below 10 and above 1 for P/A and F/P, respectively, were shown to indicate a pyrogenic source of PAHs (Budzinski et al., 1997).

### Molecular Methods and Sequence Processing

Total genomic DNA was extracted from 250 mg of sediment samples using the PowerSoil DNA isolation kit (MO-BIO Laboratories Inc.). PCR amplifications were performed using primers 27f (5′-AGRGTTTGATCMTGGCTCAG-3′) and 519r (5′-GTNTTACNGCGGCKGCTG-3′) targeting the V1–V3 region of the bacterial 16S rRNA gene (Weisburg et al., 1991), primers 344f (3′-ACGGGGYGCAGCAGGCGCGA-5′) and 915r (3′-GTGCTCCCCCGCCAATTCCT-5′) targeting the V3–V4 region of the archaeal 16S rRNA gene (Raskin et al., 1994) and primers 1560f (3′-TGGTGCATGGCCGTTCTTAGT-5′) and 2035r (3′-CATCTAAGGGCATCACAGACC-5′) targeting the VR4 region of the eukaryotic 18S rRNA gene (Hardy et al., 2010). Pyrosequencing was performed at Mr. DNA – Molecular Research (Shallowater, TX, USA), using a Roche 454 GS-FLX Titanium instrument (Roche, NJ, USA). Pyrosequencing resulted in 693687, 290816, and 110044 raw reads for bacterial, archaeal and eukaryotic datasets, respectively, (Supplementary Table S2). Raw reads from the three datasets were independently quality-based, trimmed, and aligned on the May 2013 Greengenes reference alignment for prokaryotes (McDonald et al., 2012) and SILVA 119 database for eukaryotes (Quast et al., 2013). Chimera were subsequently detected and eliminated using UCHIME (Edgar et al., 2011). All statistical analyses were performed on a random subsample of 3238, 829 and 804 sequences for bacterial, archaeal and eukaryotic datasets, respectively, corresponding to the smaller number of sequences per sample in the datasets, after trimming and quality processing. OTUs were clustered at a 90% identity threshold following the Mothur 454 Standard Operating Procedure (Schloss et al., 2009) with few modifications. The 90% threshold for OTUs is close to the taxonomic level of the family (Konstantinidis and Tiedje, 2007). This threshold was used to get sufficiently abundant OTUs for correlation analysis (i.e., enough occurrence and abundance to get statistically robust Spearman correlations). Another advantage of using this threshold is to circumvent potential taxonomic misclassifications due to sequencing anomalies (Barberán et al., 2012; Fillol et al., 2016). This resulted in 6034 bacterial OTUs, 680 archaeal OTUs and 774 eukaryotic OTUs. Taxonomic classification of each OTU was performed using the May 2013 Greengenes reference alignment for prokaryotes (McDonald et al., 2012) and the SILVA 119 database for eukaryotes (Quast et al., 2013). A representative sequence (i.e., the closest sequence of all other sequences) for each OTU was selected and a phylogenetic tree was reconstructed for each domain of life using the ARB software (Ludwig et al., 2004). Bacterial, archaeal and eukaryotic base frequency ARB filters were applied to exclude highly variable positions before sequences were added using the ARB parsimony insertion tool to the May 2013 Greengenes and SILVA 119 backbone trees, for prokaryotes and eukaryotes, respectively. Resulting phylogenetic trees were exported and used to run UniFrac weighted matrix calculation for each domain of life (Lozupone et al., 2006). Weighted UniFrac distance matrices were further used in beta-diversity analysis. We used PERMANOVA based on 1000 permutations (McArdle and Anderson, 2001) with function Adonis of the vegan package in R (Oksanen et al., 2013) to assess the source of variation in the weighted UniFrac matrices. Each dataset encompass the 42 samples of this study. The complete dataset was deposited in the NCBI Sequence Read Archive (SRA) database under study Accession no SRP063723.

### Statistical Analysis

Taking independently the Atlantic and Mediterranean regions (see Results), contaminated and non-contaminated site groups were defined by calculating and plotting Euclidean distances based on 11 PAHs as a dendrogram based on hierarchical ascendant classification (HAC, Supplementary Figure S1). Mean total PAH concentrations between contaminated and non-contaminated sites from each geographic region (i.e., Atlantic and Mediterranean regions) were compared using the Wilcoxon–Mann–Whitney *U* test.

Collinearities in independent environmental variables were tested before running permutational Manova (PERMANOVA), species abundance distribution (SAD) and network analysis. Variables with collinearity up to 0.75 were grouped together, and proxies were used as explanatory variables. Fluroranthene was the representative predictor for 10 other PAH (i.e., Fluorene, Pyrene, Anthracene, Benz[*a*]anthracene, Benzo[*a*]pyrene, Benzo[*g,h,i*]Perylene, Chrysene, Dibenz[*a,h*]Anthracene, Indeno[1,2,3,*c-d*]Pyrene and Phenanthrene) and salinity for latitude and temperature (Supplementary Table S3). Salinity and fluoranthene concentration were log transformed, and F/P ratio was squared transformed to improve the linearity and homoscedasticity of residuals.

The OTU abundance table was used to examine the SAD patterns of each OTU and partition the SAD into core and satellite microbial OTUs. For this purpose, the index of dispersion for each OTU was calculated as the ratio of the variance to the mean abundance (VMR) multiplied by the occurrence. This index was used to model whether lineages follow a Poisson distribution (i.e., stochastic distribution), falling between the 2.5 and 97.5% confidence interval of the χ^2^ distribution (Krebs, 1999).

### Co-occurrence Network Analysis

Associations between bacterial, archaeal and eukaryotic OTUs were inferred from an undirected co-occurrence network. Pairwise score between 90% core OTUs were computed using Spearman’s rank correlations. Only co-occurrences corresponding to correlations with a coefficient (rho) > 0.6 and a statistical significance (*P*-value) < 0.001 were considered for further analysis. Non-random co-occurrence patterns were tested with the checkerboard score (*C*-score) under a null model preserving site frequencies (Stone and Roberts, 1990; Gotelli and McCabe, 2002). A *C*-score calculated for each pair of microbial OTUs was compared with the *C*-score computed for 5000 randomly assembled null matrices. In order to avoid biases affecting raw *C*-score values (i.e., OTU number, abundance…), the standardized effect size (SES) was calculated (Sridhar et al., 2012). Because the *C*-score is an inverse indicator of the frequency of co-occurrence, positive SES values indicate less co-occurrence than expected by chance (i.e., predominance of segregation within communities) and *vice versa* for negative values (i.e., predominance of facilitation). If co-occurrences were not different from what was expected by chance, values of SES should fall between -2 and 2.

The network was visualized with the Gephi software (Bastian et al., 2009). Nodes represented prokaryotic and eukaryotic OTUs at 90% identity and edges represented the significant correlations between them. Network characterization for comparisons between contaminated and uncontaminated sediments was performed using a set of overall network topological indices (i.e., average node connectivity, average path length, average clustering coefficient and modularity; Newman, 2003). After module detection, each module was represented by its singular value decomposition (SVD) of abundance profile by using module eigengene analysis (Langfelder and Horvath, 2007). Module response to environmental and contaminant parameters was assessed by correlations between module eigengenes and environmental factors.

All analyses were run using the R packages vegan (Oksanen et al., 2013), igraph (Csardi and Nepusz, 2006) and WGCNA packages (Langfelder and Horvath, 2008).

## Results and Discussion

### Variation in Environmental Parameters and PAH Concentrations

**FIGURE 2:**
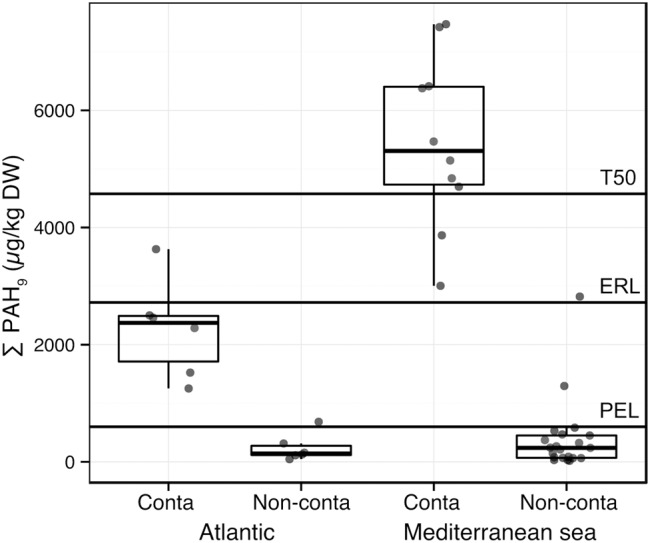
**Boxplots presenting the sum of 9 PAH concentrations (Σ PAH_9_, μg/kg DW: fluoranthene, pyrene, anthracene, benz[*a*]anthracene, benzo[*a*]pyrene, chrysene, dibenz[*a,h*]anthracene, fluorene, phenanthrene) in sediment samples from the Mediterranean Sea and Atlantic Ocean.** Plotted thresholds (full line) correspond to Sediment Quality Guidelines: Probable Effect Level (PEL), Effect Range Low (ERL), and 50% toxicity concentration (T50). Within the Atlantic and the Mediterranean Sea subsets, the means of Σ PAH_9_ are significantly different between contaminated and non-contaminated samples (*p* < 0.001, Wilcoxon–Mann–Whitney test).

Particle size distribution, %TOC, salinity, and temperature averages of the water column were significantly greater in the Mediterranean sites than in the Atlantic sites (Supplementary Figure S2). The salinity, temperature, and latitude gradients were highly correlated (Spearman rho = -0.82, *p*-value = 0.001, Supplementary Table S3). Because of the important environmental heterogeneity among the sediments from the two regions, we analyzed the PAHs contamination gradient in both regions individually. PAHs concentrations were strongly correlated to each other (rho > 0.75, *p* < 0.05, Supplementary Table S3). In order to compare the PAH concentrations to the Sediment Quality Guidelines thresholds (SQG; Buchman, 2008), which are available for 9 PAHs among the 11 PAHs of our dataset, the sums of these 9 PAHs concentrations (Σ PAH_9_) and of the corresponding SQGs were calculated (i.e., for fluoranthene, pyrene, anthracene, benz[*a*]anthracene, benzo[*a*]pyrene, chrysene, dibenz[*a,h*]anthracene, fluorene, phenanthrene; **Figure 2**). The SQG used are the following: PEL, ERL, which are low trigger concentrations and T50 (50% toxicity concentration) values.

The HAC analysis confirmed the *a priori* classification (i.e., pre-sampling classification) for most of the samples, and particularly for Mediterranean samples (Supplementary Figure S1). **Figure 2** shows the differences of mean Σ PAH_9_ concentrations between Mediterranean and Atlantic regions. Contaminated samples determined using the HAC analysis exhibited significantly higher mean PAH concentrations in both Mediterranean and Atlantic regions (**Figure 2**). However, the difference between contaminated and non-contaminated samples was much greater in the Mediterranean sites. When comparing mean Σ PAH_9_ concentrations, PEL, ERL, and T50 separated Mediterranean contaminated sites from non-contaminated sites, while only PEL could be a threshold between contaminated and non-contaminated Atlantic sites (**Figure 2**). SQGs were originally defined regarding macrofauna ecotoxicological assays (Buchman, 2008), and little is known about their reliability for the determination of PAHs toxicity on microorganisms (Sun et al., 2012). However, bacterial community structure from chronically contaminated estuarine sediment was previously found to respond to low trigger concentrations (Sun et al., 2012). These structure modifications were mainly related to change in core OTU abundances (Sun et al., 2013).

We used diagnosis ratios of PAH concentrations (i.e., fluoranthene to pyrene (F/P) and phenanthrene to anthracene (P/A)) to get an indication of the dominant source of contamination by PAHs (i.e., pyrogenic versus petrogenic; Budzinski et al., 1997; Neff et al., 2005). Most of the samples, from both the Mediterranean and the Atlantic regions, exhibited P/A values lower than 10 and F/P values above 1, indicating a pyrogenic contamination (Supplementary Figure S3, Supplementary Table S1). In contrast, none of the sites exhibited clear petrogenic contamination according to these ratios. PAHs derived from combustion in coastal sediments occurs through atmospheric deposition (Lipiatou et al., 1997; Golomb et al., 2001), which constitutes a chronic PAH input since the Industrial Revolution (Lima et al., 2003; Azoury et al., 2013). Considering the diagnosis ratios, we could therefore infer that the samples analyzed here were thus facing mainly chronic contamination by pyrolytic PAHs.

### Partitioning the Core and Satellite Communities

Traditionally in macro-ecology, the SAD in spatially or temporally separated sites can be divided into two groups: the core species/taxa, which are widely distributed and generally abundant, and the satellite species/taxa, which mostly occur in low abundance and at a reduced number of sites (Hanski, 1982; Magurran and Henderson, 2003; Ulrich and Zalewski, 2006). Previous studies showed that this division of the SAD was not artifactual and demonstrated that environmental factors shaped the relative abundance of the core species/taxa, whereas random dispersal mostly explained satellite species/taxa community assembly (Magurran and Henderson, 2003; Ulrich and Zalewski, 2006; Magurran, 2007). The concept has been reinforced in subsequent studies, which suggested that relevant aspects of the SAD could be overlooked without such a partition (Ulrich and Ollik, 2004; Gray et al., 2005; Dolan et al., 2009). Despite a growing number of surveys interested by rare or core communities in microbial ecology (Galand et al., 2009; Pedrós-Alió, 2012; Hugoni et al., 2013; Cariveau et al., 2014), the mechanistic framework developed by Magurran and Henderson (2003) to partition the SAD has been very rarely used (Fillol et al., 2016).

**FIGURE 3:**
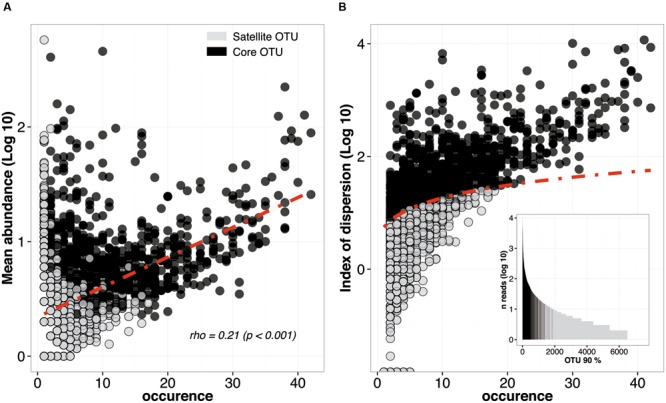
**Species abundance distribution (SAD) pattern of microbial OTUs in the 42 libraries analyzed. (A)** Occurrence of OTU (number of studies in which a given OTU was found) plotted against its average abundance across these studies. The dotted line depicts the positive correlation between abundance and occurrence. Black and gray dots represent core and satellite OTUs, respectively. **(B)** Occurrence of each microbial OTU plotted against its dispersion index. The line depicts the 2.5% confidence limit of the χ^2^ distribution: lineages falling bellow this line follow a Poisson distribution and are randomly dispersed in space. A rank abundance plot of all the OTUs is represented in order to indicate the ranking of the core OTUs.

For this purpose, 7488 microbial OTUs (6034 bacteria, 680 archaea, and 774 eukaryotes OTUs at 90% cutoff) from the 42 sediments sampled in this study were analyzed in an abundance-*vs.*-occurrence plot (**Figure 3A**). In agreement with one of the most robust trend in macro-ecology (Gaston et al., 2000), we found a significant and positive relationship between the mean abundance and the occurrence. However, due to a high number of OTUs, the typical discontinuity that separates core from satellite OTUs was not appreciable (**Figure 3A**). In order to partition the SAD, we compared the index of dispersion for each OTU to a model assuming a stochastic distribution (Poisson model) falling between the 2.5% and 97.5% confidence limit of the χ^2^ distribution (Krebs, 1999). Overall, 859 OTUs (11% of the initial number of OTUs) were found to form the core microbial community of coastal sediments (**Figure 3B**). They represented 80% of the reads. In contrast, the remaining 89% of the OTUs fell below the 2.5% confidence limit line, indicating random distribution through sediment samples (**Figure 3B**). The ranking abundance plot confirmed that this method represent a tractable and reproducible approach to partition the SAD of microbial communities (**Figure 3B**). It also highlights the fact that the definition of core or satellite OTUs should not be only based on abundance or occurrence/permanence but on both, as suggested by Hubbell (2001).

### Composition and Sensitivity of Core and Satellite Community Members to Environmental Parameters and Hydrocarbon Contamination

**FIGURE 4:**
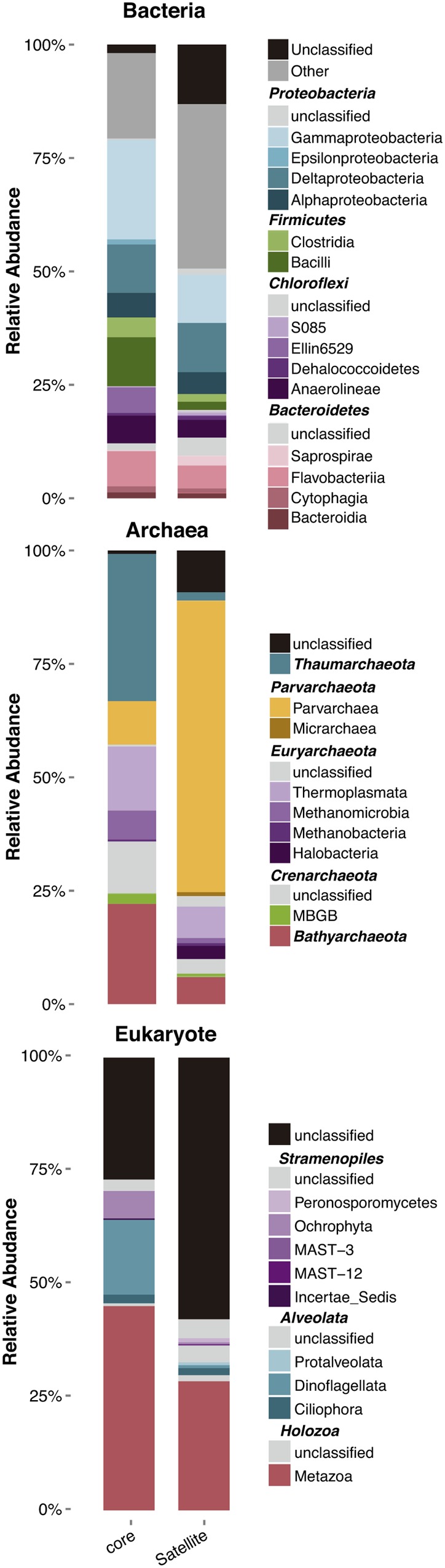
**Relative abundance of the different microbial taxa in core and satellite groups at the taxonomic level of the class.** Only Classes belonging to Phyla representing more than 5% of total relative abundance were represented. The whole taxonomy of both core and satellite OTUs is available in .csv files within the Supplementary Material.

Core and satellite community compositions were found to differ significantly for the three domains of life (**Figure 4**). A high percentage of satellite OTUs remained unclassified at the phylum level, particularly for eukaryotes with more than 50% of the OTU that could not be affiliated to any taxa. For bacteria, the core community was dominated by OTUs belonging to the *Gammaproteobacteria* (16% of the total abundance) and *Deltaproteobacteria* (9%), which are known to be dominant clades in marine sediments (both contaminated and pristine) and to encompass a large number of hydrocarbon-degrader strains (Paissé et al., 2008; Zhang et al., 2008; Rojo, 2009; Kostka et al., 2011; Acosta-González et al., 2013; Sun et al., 2013). The dominance of *Deltaproteobacteria* OTUs within the core community is in agreement with results found along a pollution gradient in estuarine sediments (Sun et al., 2013). This class includes most of the sulfate-reducing genera, which play a crucial role in the anaerobic degradation of organic matter (Leloup et al., 2009), but also in hydrocarbon degradation (Suárez-Suárez et al., 2011; Stauffert et al., 2014a). Archaeal core community was the most divergent from the satellite community, with domination by OTUs belonging to the *Thaumarchaeota, Bathyarchaeota* and *Thermoplasmata* (**Figure 4**). The latter groups, which represented 22 and 14% of total abundance, respectively, were found to be part of the core microbiota in a meta-analysis regrouping more than 200 sediment studies (Fillol et al., 2016). Both archaeal groups seemed to form specific consortia in sediment ecosystems, suggesting a potentially relevant trophic connection related to the degradation of aromatic compounds and detrital proteins (Lloyd et al., 2013; Meng et al., 2014; Fillol et al., 2016). In contrast, distribution and potential functions of the *Parvarchaeota* (previously known as *Haloarchaea*), which represented 64% of the total abundance in the archaeal satellite community, are currently unknown.

For eukaryote a large proportion of the OTUs of the core and satellite communities remained unclassified (i.e., 25% and 57%, respectively), highlighting our ignorance about eukaryotic communities inhabiting coastal sediments. With a growing number of environmental surveys investigating the structure and diversity of eukaryotic communities using the 18S rDNA gene and NGS (next generation sequencing), this result underpinned the urge for the development of eukaryotic taxonomy databases similar to those for prokaryotes. The remaining OTUs were dominated both in the core and satellite communities by members of the *Holozoa* kingdom and the *Alveolata* superphylum with 45 and 18% of the total abundance, respectively.

We conducted PERMANOVA analyses in order to identify whether the core or the satellite community would show the highest sensitivity to changes in environmental and contaminant variables (**Table 1**). As expected, we observed a strong filtering effect on the most abundant and frequent OTUs forming the core community, with salinity (associated with latitude and temperature) representing the main environmental parameters driving the community composition. %TOC and particle size distribution (% > 63 μm) together explained an additional 10% of the variance in community composition, while fluoranthene explained only 2% of this variance. The response to the measured predictors was weak for the OTUs forming the satellite community, with only 2% of the overall variance explained by salinity. This result may indicate a minimal influence of satellite OTUs on biogeochemical cycles as previously postulated (Gobet et al., 2012), and contrary to core OTUs that are thought to contribute actively to the biomass and the nutrient cycling within the community (Campbell et al., 2011; Gaidos et al., 2011; Pedrós-Alió, 2012). However, *Gammaproteobacteria* and *Deltaproteobacteria*, which encompass many species able to degrade hydrocarbons (Prince et al., 2010), were found in similar proportions in the rare and core communities (**Figure 4**), suggesting the potential role of the rare benthic biosphere in hydrocarbon degradation. Bacterial hydrocarbon-degraders, such as *Cycloclasticus*, were depicted as members of the rare community in non-polluted environment, and became dominant in the presence of hydrocarbon contamination (Teira et al., 2007; Sauret et al., 2014). This fluctuating state of specialized taxa from the rare community could facilitate the adaptation of the whole community to chronic hydrocarbon contamination. Overall, despite the predominant response of the core community to environmental changes, we cannot exclude the potentially central role of members of the rare community in chronically hydrocarbon-polluted sediments.

**Table 1:** Results of the PERMANOVA analysis for the estimation of the relative contribution of environmental parameters to the phylogenetic community composition of the core and satellite microbial communities.

PERMANOVA (*n* = 32)	Core	Satellite
Fluoranthene	2%^∗^	
Phenanthrene/Anthracene (P/A)		
Fluoranthene to pyrene (F/P)		
Salinity	11%^∗∗∗^	2%^∗^
Particle size distribution (% > 63 μm)	5%^∗∗^	
% Total Organic Carbon	5%^∗∗^	

*Fluoranthene was a proxy for 10 other PAH (i.e., fluoranthene, pyrene, anthracene, benz[*a*]anthracene, benzo[*a*]pyrene, chrysene, dibenz[*a,h*]anthracene, fluorene, phenanthrene), salinity for latitude and temperature. ^∗^*p* < 0.05, ^∗∗^*p* < 0.01, ^∗∗∗^*p* < 0.001.*

### Response of Microbial Consortia and Network Properties to PAH Contaminations

**FIGURE 5:**
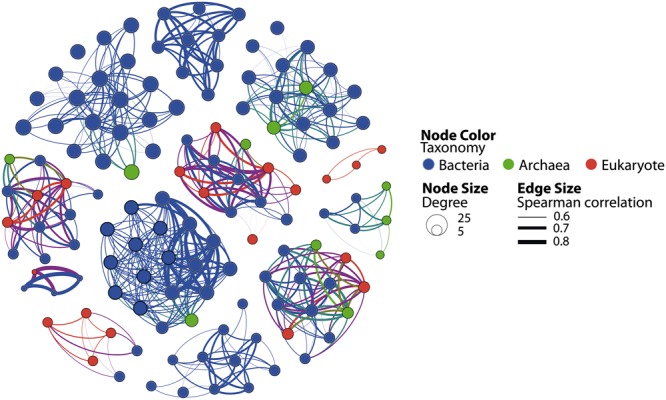
**Co-occurrence OTU network based on correlation analysis.** Each node denotes a microbial OTU 90%. Node size is proportional to the degree (i.e., the number of links from this node to any other node) and node color denotes taxonomic classification. Edge lines between nodes represent significant co-occurrence relationships. Edge size indicates the strength of Spearman correlation among nodes.

**FIGURE 6:**
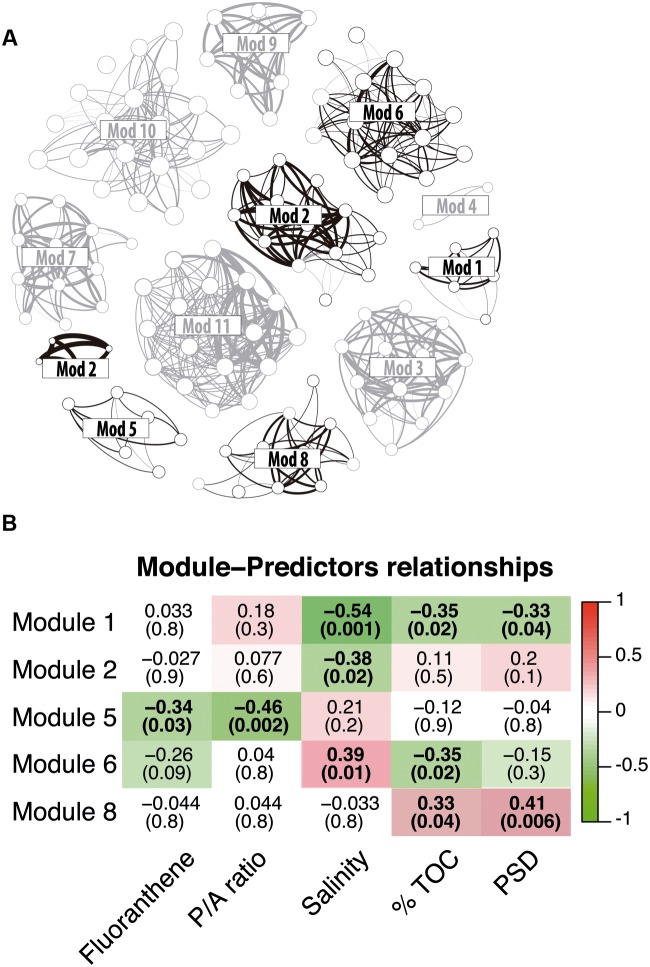
**(A)** Same network as in **Figure 5**, but modules are indicated. Modules significantly related to environmental parameters are colored in black. **(B)** The correlations between module eigengenes and environmental traits in the core community network. Only module significantly related to environmental parameters and contaminants are shown. The color of each plot indicates the correlation between corresponding module eigengene and environmental trait. Red color means highly positive correlation and green color means highly negative correlation. The numbers in each plot are the correlation coefficient (r) and significance (p) in parentheses. Correlations with *p* > 0.05 are in bold.

**FIGURE 7:**
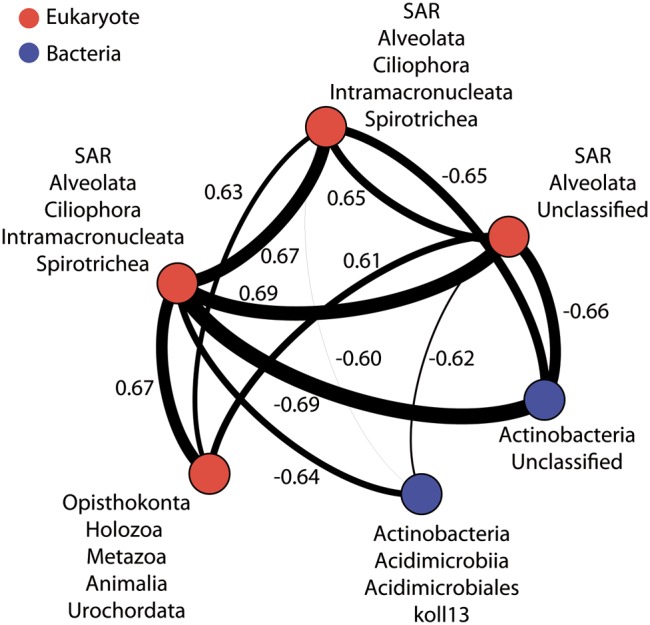
**Co-occurrence OTU network for module 5.** Edge size indicates the number of connections (degree). Node size is proportional to the degree (i.e., the number of links from this node to any other node) and node color denotes taxonomic classification. Edge lines between nodes represent significant co-occurrences relationships. Values indicated Spearman correlations.

As illustrated by recent studies, microbial co-occurrence patterns can help to unveil ecologically meaningful interactions between taxa (Horner-Devine et al., 2007; Steele et al., 2011; Fillol et al., 2016) and network properties can be used to study community stability in altered conditions (Zhou et al., 2011; Deng et al., 2012; Faust and Raes, 2012; Sun et al., 2013). To get a first insight in the potential microbial consortia affected by PAH contaminations and the potential effect of these contaminants on the core community integrity, we constructed a co-occurrence network based on strong and significant Spearman correlations. The dataset containing core OTUs was filtered for optimizing network sensitivity and specificity. The final network included 131 nodes and 566 edges (**Figure 5**). We used the SES of the *C*-score metric to assess the existence of non-random co-occurrence patterns in the network (Gotelli and McCabe, 2002; Horner-Devine et al., 2007; López et al., 2013). We observed a SES value of 2.56 (*P* < 0.001, *C*-score_norm_ = 0.88) indicating non-random network structure. The network was largely dominated by bacterial OTUs, which represented 76% of the nodes (**Figure 5**). *Eukarya* and *Archaea* represented 15 and 9% of the nodes, respectively. Correlations between domains were common within the network although intra-domain connections tended to be more numerous. These co-occurrences might reflect OTUs performing similarly or harboring complementary functions, but they may also be caused by shared preferred environmental conditions (Steele et al., 2011; Deng et al., 2012). Network approaches showed that co-occurring OTUs are often organized into groups, or modules, of functional significance (Chaffron et al., 2010; Barberán et al., 2012; Vick-Majors et al., 2014). Modularity analysis revealed a complex structure composed of 11 modules (**Figure 6A**). Modules are groups of highly connected OTUs within the group but with very few connections outside the group. **Figure 6B** shows the response of modules to environment changes, as assessed by means of correlations between modules and environmental parameters. Five of the 11 modules detected were significantly correlated with the environmental parameters measured in this study (**Figure 6B**). Modules 1, 2, and 6 were correlated to salinity (and associated latitude and temperature), confirming the strong effect of these variables on microbial consortia. In agreement with previous works (Chaffron et al., 2010; Freilich et al., 2010; Faust and Raes, 2012), modules could be considered as ecological and/or functional niches as suggested by modules 1, 2 and 6, which represented low salinity/high latitude and high salinity/low latitude sub-networks, respectively. Module 5 was the only microbial consortium responding (negatively) to fluoranthene and associated PAH concentrations, indicating that PAH contaminations might have a negative impact on the members of this module. This module was composed of four eukaryotic and two bacterial OTUs connected by an unusual number of negative correlations between OTUs belonging to *Actinobacteria* and OTUs belonging to the *Alveolata* taxa (**Figure 7**), suggesting predator – prey relationships (Faust and Raes, 2012). This assumption is in agreement with the fact that Alveolates, and more particularly Ciliates, feed on a variety of prey but that many ciliate groups are bacterivores (Meng et al., 2012).

Comparing network properties and overall topology can be used to study community stability/vulnerability in altered conditions. In order to assess the influence of hydrocarbon contaminants on network properties, we split the core OTU abundance table based on the classification provided by the HAC (Supplementary Figure S2) and constructed separated microbial networks for non-contaminated and contaminated sediment communities (**Table 2**). Non-contaminated and contaminated networks were generated for the whole dataset and for each region (i.e., Mediterranean and Atlantic) in order to limit the influence of environmental variables (i.e., salinity, temperature, latitude, PSD and %TOC, which were significantly different between Mediterranean and Atlantic regions). Network comparisons are commonly achieved by calculating the average for each node of four network indices (i) connectivity or degree distribution, which represent the number of edges of a node toward other nodes; (ii) path length, which is the shortest path between two nodes; (iii) the clustering coefficient, which describes how well a node is connected to its neighbors; (iv) modularity, a measure of the structuration of the network into modules (Deng et al., 2012). **Table 2** showed that the three pairs of networks calculated here presented the typical topology for microbial networks (Chaffron et al., 2010; Steele et al., 2011; Barberán et al., 2012; Deng et al., 2012): scale-free (i.e., node connectivity distribution not different from a power law model, data not shown), small-world (clustering coefficient ranging from 0.56 to 0.89 and average path from 2.22 to 2.75) and modular (modularity ranging from 0.72 to 0.86). Overall and except for the Atlantic samples, significant differences between contaminated and non-contaminated networks were detected in terms of average connectivity, average shortest path and average clustering coefficient (**Table 2**). These results indicated that hydrocarbon contaminations might have a significant and negative effect on the interactions among microbial taxa in coastal sediments. Indeed, the higher values for average shortest path in contaminated networks may imply lower efficiency and speed in the transmission of information, energy or material in the system, potentially altering the response of the microbial community to environmental perturbations (Zhou et al., 2010; Deng et al., 2012). In addition, the lower connectivity and lower average clustering coefficient in contaminated networks may make the networks less resistant to change (Albert et al., 2000; Montoya et al., 2006). This may also indicate a potential perturbation of the network and the loss of important community members. A lower clustering coefficient is indeed an indication of the loss of highly connected nodes (i.e., hubs), which have been related to the concept of keystone species (Paine, 1969; Steele et al., 2011). Such keystone species loss could cause a greater fragility of the network, which ultimately could lead to its fragmentation and to secondary extinctions of species relying on the hubs (Solé and Montoya, 2001).

**Table 2:** Topological properties of the networks generated for core communities in contaminated and non-contaminated samples.

Network properties	Ctot-prist	Ctot-cont	Cmed-prist	Cmed-cont	Catl-prist	Catl-cont
N° of original OTUs	322	312	216	197	167	166
N° of OTU in the network	126	133	98	99	76	78
Average Degree (connectivity)	9.2^a^	8.6^a^	6.8^a^	5.1^a^	5	5.1
Average clustering coefficient	0.89^a^	0.74^a^	0.62^a^	0.56^a^	0.64	0.63
Modularity	0.86	0.84	0.74	0.72	0.73	0.75
Average shortest path (geodesic distance)	1.31^a^	1.75^a^	1.22^a^	1.70^a^	1.24	1.25

*Ctot, Cmed and Catl stand for whole data set, Mediterranean samples, Atlantic samples, respectively. Prist and cont stand for pristine and contaminated samples. ^a^Significant difference (*P* < 0.001) between values from non contaminated and contaminated networks.*

Altogether, these results suggested that hydrocarbon contaminations might have altered the microbial network in coastal sediments, rendering the microbial communities inhabiting these habitats more vulnerable to environmental perturbations.

## Author Contributions

J-CA, JG, and RD conceived and designed the study. MJ ran the experiments. J-CA and MJ ran the data analysis. J-FG, JT, OB, and HA provided sediment samples, chemical and physical data. J-CA and MJ prepared the manuscript.

## Conflict of Interest Statement

The authors declare that the research was conducted in the absence of any commercial or financial relationships that could be construed as a potential conflict of interest.

## References

[B1] Acosta-González A. Rosselló-Móra R. Marqués S. (2013). Characterization of the anaerobic microbial community in oil-polluted subtidal sediments: aromatic biodegradation potential after the Prestige oil spill. *Environ. Microbiol.* 15 77–92. 10.1111/j.1462-2920.2012.02782.x 22626032

[B2] Albert R. Jeong H. Barabási A.-L. (2000). Error and attack tolerance of complex networks. *Nature* 406 378–382. 10.1038/35019019 10935628

[B3] Azoury S. Tronczyñski J. Chiffoleau J.-F. Cossa D. Nakhlé K. Schmidt S. (2013). Historical records of mercury, lead, and polycyclic aromatic hydrocarbons depositions in a dated sediment core from the eastern mediterranean. *Environ. Sci. Technol.* 47 7101–7109. 10.1021/es4005637 23725373

[B4] Barberán A. Bates S. T. Casamayor E. O. Fierer N. (2012). Using network analysis to explore co-occurrence patterns in soil microbial communities. *ISME J.* 6 343–351. 10.1038/ismej.2011.119 21900968 PMC3260507

[B5] Bastian M. Heymann S. Jacomy M. (2009). Gephi: an open source software for exploring and manipulating networks. *ICWSM* 8 361–362.

[B6] Ben Said O. Goñi-Urriza M. El Bour M. Aissa P. Duran R. (2010). Bacterial community structure of sediments of the Bizerte lagoon (Tunisia), a southern mediterranean coastal anthropized lagoon. *Microb. Ecol.* 59 445–456. 10.1007/s00248-009-9585-x 19789910

[B7] Buchman M. F. (2008). *Screening Quick Reference Tables (SQuiRTs).* Seattle, WA: US National Oceanic and Atmospheric Administration, Office of Response and Restoration Division.

[B8] Budzinski H. Jones I. Bellocq J. Piérard C. Garrigues P. (1997). Evaluation of sediment contamination by polycyclic aromatic hydrocarbons in the Gironde estuary. *Mar. Chem.* 58 85–97. 10.1016/S0304-4203(97)00028-5

[B9] Campbell B. J. Yu L. Heidelberg J. F. Kirchman D. L. (2011). Activity of abundant and rare bacteria in a coastal ocean. *Proc. Natl. Acad. Sci.* 108 12776–12781. 10.1073/pnas.1101405108 21768380 PMC3150899

[B10] Cariveau D. P. Elijah Powell J. Koch H. Winfree R. Moran N. A. (2014). Variation in gut microbial communities and its association with pathogen infection in wild bumble bees (*Bombus*). *ISME J.* 8 2369–2379. 10.1038/ismej.2014.68 24763369 PMC4260702

[B11] Chaffron S. Rehrauer H. Pernthaler J. Mering C. (2010). A global network of coexisting microbes from environmental and whole-genome sequence data. *Genome Res.* 20 947–959. 10.1101/gr.104521.109 20458099 PMC2892096

[B12] Csardi G. Nepusz T. (2006). The igraph software package for complex network research. *InterJournal Complex Syst.* 1695 1–9.

[B13] Deng Y. Jiang Y.-H. Yang Y. He Z. Luo F. Zhou J. (2012). Molecular ecological network analyses. *BMC Bioinformatics* 13:113 10.1186/1471-2105-13-113 PMC3428680 22646978

[B14] Dias A. C. F. Dini-Andreote F. Taketani R. G. Tsai S. M. Azevedo J. L. Melo I. S. (2011). Archaeal communities in the sediments of three contrasting mangroves. *J. Soils Sediments* 11 1466–1476. 10.1007/s11368-011-0423-7

[B15] Dolan J. R. Ritchie M. E. Tunin-Ley A. Pizay M.-D. (2009). Dynamics of core and occasional species in the marine plankton: tintinnid ciliates in the north-west mediterranean sea. *J. Biogeogr.* 36 887–895. 10.1111/j.1365-2699.2008.02046.x

[B16] Dunne J. A. Williams R. J. Martinez N. D. (2002a). Food-web structure and network theory: the role of connectance and size. *Proc. Natl. Acad. Sci.* 99 12917–12922. 10.1073/pnas.192407699 12235364 PMC130560

[B17] Dunne J. A. Williams R. J. Martinez N. D. (2002b). Network structure and biodiversity loss in food webs: robustness increases with connectance. *Ecol. Lett.* 5 558–567. 10.1046/j.1461-0248.2002.00354.x

[B18] Edgar R. C. Haas B. J. Clemente J. C. Quince C. Knight R. (2011). UCHIME improves sensitivity and speed of chimera detection. *Bioinformatics* 27 2194–2200. 10.1093/bioinformatics/btr381 21700674 PMC3150044

[B19] European Environment Agency (2006). *Priority Issues in the Mediterranean Environment.* Copenhagen: European Environmental Agency.

[B20] Faust K. Raes J. (2012). Microbial interactions: from networks to models. *Nat. Rev. Microbiol.* 10 538–550. 10.1038/nrmicro2832 22796884

[B21] Fernández-Luqueño F. Valenzuela-Encinas C. Marsch R. Martínez-Suárez C. Vázquez-Núñez E. Dendooven L. (2010). Microbial communities to mitigate contamination of PAHs in soil—possibilities and challenges: a review. *Environ. Sci. Pollut. Res.* 18 12–30. 10.1007/s11356-010-0371-6 20623198

[B22] Fillol M. Auguet J.-C. Casamayor E. O. Borrego C. M. (2016). Insights in the ecology and evolutionary history of the miscellaneous crenarchaeotic group lineage. *ISME J.* 10 665–677. 10.1038/ismej.2015.143 26284443 PMC4817671

[B23] Freilich S. Kreimer A. Meilijson I. Gophna U. Sharan R. Ruppin E. (2010). The large-scale organization of the bacterial network of ecological co-occurrence interactions. *Nucleic Acids Res.* 38 3857–3868. 10.1093/nar/gkq118 20194113 PMC2896517

[B24] Gaidos E. Rusch A. Ilardo M. (2011). Ribosomal tag pyrosequencing of DNA and RNA from benthic coral reef microbiota: community spatial structure, rare members and nitrogen-cycling guilds. *Environ. Microbiol.* 13 1138–1152. 10.1111/j.1462-2920.2010.02392.x 21176054

[B25] Galand P. E. Casamayor E. O. Kirchman D. L. Lovejoy C. (2009). Ecology of the rare microbial biosphere of the arctic ocean. *Proc. Natl. Acad. Sci.* 106 22427–22432. 10.1073/pnas.0908284106 20018741 PMC2796907

[B26] Gaston K. J. Blackburn T. M. Greenwood J. J. D. Gregory R. D. Quinn R. M. Lawton J. H. (2000). Abundance–occupancy relationships. *J. Appl. Ecol.* 37 39–59. 10.1046/j.1365-2664.2000.00485.x

[B27] Gobet A. Böer S. I. Huse S. M. van Beusekom J. E. E. Quince C. Sogin M. L. (2012). Diversity and dynamics of rare and of resident bacterial populations in coastal sands. *ISME J.* 6 542–553. 10.1038/ismej.2011.132 21975598 PMC3280144

[B28] Golomb D. Barry E. Fisher G. Varanusupakul P. Koleda M. Rooney T. (2001). Atmospheric deposition of polycyclic aromatic hydrocarbons near New England coastal waters. *Atmos. Environ.* 35 6245–6258. 10.1016/S1352-2310(01)00456-3

[B29] Gotelli N. J. McCabe D. J. (2002). Species co-occurrence: a meta-analysis of J. M. diamond’s assembly rules model. *Ecology* 83 2091–2096. 10.1890/0012-9658(2002)083[2091:SCOAMA]2.0.CO;2

[B30] Gray J. S. Bjørgesæter A. Ugland K. I. (2005). The impact of rare species on natural assemblages. *J. Anim. Ecol.* 74 1131–1139. 10.1111/j.1365-2656.2005.01011.x

[B31] Hanski I. (1982). Dynamics of regional distribution: the core and satellite species hypothesis. *Oikos* 38 210–221. 10.2307/3544021

[B32] Hardy C. M. Krull E. S. Hartley D. M. Oliver R. L. (2010). Carbon source accounting for fish using combined DNA and stable isotope analyses in a regulated lowland river weir pool. *Mol. Ecol.* 19 197–212. 10.1111/j.1365-294X.2009.04411.x 19912537

[B33] Head I. M. Jones D. M. Röling W. F. (2006). Marine microorganisms make a meal of oil. *Nat. Rev. Microbiol.* 4 173–182. 10.1038/nrmicro1348 16489346

[B34] Horner-Devine M. C. Silver J. M. Leibold M. A. Bohannan B. J. M. Colwell R. K. Fuhrman J. A. (2007). A comparison of taxon co-occurrence patterns for macro- and microorganisms. *Ecology* 88 1345–1353. 10.1890/06-0286 17601127

[B35] Hubbell S. P. (2001). *The Unified Neutral Theory of Biodiversity and Biogeography (MPB-32).* Princeton, NJ: Princeton University Press.

[B36] Hugoni M. Taib N. Debroas D. Domaizon I. Dufournel I. J. Bronner G. (2013). Structure of the rare archaeal biosphere and seasonal dynamics of active ecotypes in surface coastal waters. *Proc. Natl. Acad. Sci.* 110 6004–6009. 10.1073/pnas.1216863110 23536290 PMC3625260

[B37] Kimes N. E. Callaghan A. V. Suflita J. M. Morris P. J. (2014). Microbial transformation of the deepwater horizon oil spill–past, present, and future perspectives. *Front. Microbiol.* 5:603 10.3389/fmicb.2014.00603 PMC4235408 25477866

[B38] King G. M. Kostka J. E. Hazen T. C. Sobecky P. A. (2015). Microbial responses to the deepwater horizon oil spill: from coastal wetlands to the deep sea. *Annu. Rev. Mar. Sci.* 7 377–401. 10.1146/annurev-marine-010814-015543 25251273

[B39] Konstantinidis K. T. Tiedje J. M. (2007). Prokaryotic taxonomy and phylogeny in the genomic era: advancements and challenges ahead. *Curr. Opin. Microbiol.* 10 504–509. 10.1016/j.mib.2007.08.006 17923431

[B40] Kostka J. E. Prakash O. Overholt W. A. Green S. J. Freyer G. Canion A. (2011). Hydrocarbon-degrading bacteria and the bacterial community response in gulf of mexico beach sands impacted by the deepwater horizon oil spill. *Appl. Environ. Microbiol.* 77 7962–7974. 10.1128/AEM.05402-11 21948834 PMC3208977

[B41] Krebs C. J. (1999). *Ecological Methodology.* Menlo Park, CA: Benjamin/Cummings.

[B42] Langfelder P. Horvath S. (2007). Eigengene networks for studying the relationships between co-expression modules. *BMC Syst. Biol.* 1:54 10.1186/1752-0509-1-54 PMC2267703 18031580

[B43] Langfelder P. Horvath S. (2008). WGCNA: an R package for weighted correlation network analysis. *BMC Bioinformatics* 9:1 10.1186/1471-2105-9-559 PMC2631488 19114008

[B44] Leloup J. Fossing H. Kohls K. Holmkvist L. Borowski C. Jørgensen B. B. (2009). Sulfate-reducing bacteria in marine sediment (Aarhus Bay, Denmark): abundance and diversity related to geochemical zonation. *Environ. Microbiol.* 11 1278–1291. 10.1111/j.1462-2920.2008.01855.x 19220398

[B45] Lima A. L. C. Eglinton T. I. Reddy C. M. (2003). High-resolution record of pyrogenic polycyclic aromatic hydrocarbon deposition during the 20th century. *Environ. Sci. Technol.* 37 53–61. 10.1021/es025895p 12542290

[B46] Lipiatou E. Tolosa I. Simó R. Bouloubassi I. Dachs J. Marti S. (1997). Mass budget and dynamics of polycyclic aromatic hydrocarbons in the mediterranean sea. Deep sea res. part II top. *Stud. Oceanogr.* 44 881–905. 10.1016/S0967-0645(96)00093-8

[B47] Lloyd K. G. Schreiber L. Petersen D. G. Kjeldsen K. U. Lever M. A. Steen A. D. (2013). Predominant archaea in marine sediments degrade detrital proteins. *Nature* 496 215–218. 10.1038/nature12033 23535597

[B48] López R. P. Valdivia S. Rivera M. L. Rios R. S. (2013). Co-occurrence patterns along a regional aridity gradient of the subtropical andes do not support stress gradient hypotheses. *PLoS ONE* 8:e58518 10.1371/journal.pone.0058518 PMC3591305 23505527

[B49] Lozupone C. Hamady M. Knight R. (2006). UniFrac–an online tool for comparing microbial community diversity in a phylogenetic context. *BMC Bioinformatics* 7:371 10.1186/1471-2105-7-371 PMC1564154 16893466

[B50] Ludwig W. Strunk O. Westram R. Richter L. Meier H. Yadhukumar (2004). ARB: a software environment for sequence data. *Nucleic Acids Res.* 32 1363–1371. 10.1093/nar/gkh293 14985472 PMC390282

[B51] Magurran A. E. (2007). Species abundance distributions over time. *Ecol. Lett.* 10 347–354. 10.1111/j.1461-0248.2007.01024.x 17498133

[B52] Magurran A. E. Henderson P. A. (2003). Explaining the excess of rare species in natural species abundance distributions. *Nature* 422 714–716. 10.1038/nature01547 12700760

[B53] McArdle B. H. Anderson M. J. (2001). Fitting multivariate models to community data: a comment on distance-based redundancy analysis. *Ecology* 82 290–297. 10.1890/0012-9658(2001)082[0290:FMMTCD]2.0.CO;2

[B54] McDonald D. Price M. N. Goodrich J. Nawrocki E. P. DeSantis T. Z. Probst A. (2012). An improved Greengenes taxonomy with explicit ranks for ecological and evolutionary analyses of bacteria and archaea. *ISME J.* 6 610–618. 10.1038/ismej.2011.139 22134646 PMC3280142

[B55] Meng J. Xu J. Qin D. He Y. Xiao X. Wang F. (2014). Genetic and functional properties of uncultivated MCG archaea assessed by metagenome and gene expression analyses. *ISME J.* 8 650–659. 10.1038/ismej.2013.174 24108328 PMC3930316

[B56] Meng Z. Xu K. Dai R. Lei Y. (2012). Ciliate community structure, diversity and trophic role in offshore sediments from the yellow sea. *Eur. J. Protistol.* 48 73–84. 10.1016/j.ejop.2011.08.001 22030401

[B57] Montoya J. M. Pimm S. L. Solé R. V. (2006). Ecological networks and their fragility. *Nature* 442 259–264. 10.1038/nature04927 16855581

[B58] Nealson K. H. (1997). Sediment bacteria: who’s there, what are they doing, and what’s new? *Annu. Rev. Earth Planet. Sci.* 25 403–434. 10.1146/annurev.earth.25.1.403 11540735

[B59] Neff J. M. Stout S. A. Gunster D. G. (2005). Ecological risk assessment of polycyclic aromatic hydrocarbons in sediments: identifying sources and ecological hazard. *Integr. Environ. Assess. Manag.* 1 22–33. 10.1897/IEAM_2004a-016.1 16637144

[B60] Newman M. (2003). The structure and function of complex networks. *SIAM Rev.* 45 167–256. 10.1137/S003614450342480

[B61] Nogales B. Aguiló-Ferretjans M. M. Martín-Cardona C. Lalucat J. Bosch R. (2007). Bacterial diversity, composition and dynamics in and around recreational coastal areas. *Environ. Microbiol.* 9 1913–1929. 10.1111/j.1462-2920.2007.01308.x 17635539

[B62] Nogales B. Lanfranconi M. P. Piña-Villalonga J. M. Bosch R. (2011). Anthropogenic perturbations in marine microbial communities. *FEMS Microbiol. Rev.* 35 275–298. 10.1111/j.1574-6976.2010.00248.x 20738403

[B63] Oil in the Sea III (2003). *Oil in the Sea III: Inputs, Fates, and Effects.* Washington, DC: National Academies Press. 25057607

[B64] Oksanen J. Blanchet F. G. Kindt R. Legendre P. Minchin P. R. O’Hara R. B. (2013). Package “vegan.”. *R Packag Ver.* 254 20–28.

[B65] Olesen J. M. Bascompte J. Dupont Y. L. Jordano P. (2007). The modularity of pollination networks. *Proc. Natl. Acad. Sci.* 104 19891–19896. 10.1073/pnas.0706375104 18056808 PMC2148393

[B66] Paine R. T. (1969). A note on trophic complexity and community stability. *Am. Nat.* 103 91–93. 10.1086/282586

[B67] Paissé S. Coulon F. Goñi-Urriza M. Peperzak L. McGenity T. J. Duran R. (2008). Structure of bacterial communities along a hydrocarbon contamination gradient in a coastal sediment. *FEMS Microbiol. Ecol.* 66 295–305. 10.1111/j.1574-6941.2008.00589.x 18803671

[B68] Païssé S. Goñi-Urriza M. Coulon F. Duran R. (2010). How a bacterial community originating from a contaminated coastal sediment responds to an oil input. *Microb. Ecol.* 60 394–405. 10.1007/s00248-010-9721-7 20652237

[B69] Pedrós-Alió C. (2012). The rare bacterial biosphere. *Annu. Rev. Mar. Sci* 4 449–466. 10.1146/annurev-marine-120710-100948 22457983

[B70] Prince R. C. Gramain A. McGenity T. J. (2010). “Prokaryotic hydrocarbon degraders,” in *Handbook of Hydrocarbon and Lipid Microbiology* ed. Timmis K. N. (Berlin: Springer) 1669–1692.

[B71] Quast C. Pruesse E. Yilmaz P. Gerken J. Schweer T. Yarza P. (2013). The SILVA ribosomal RNA gene database project: improved data processing and web-based tools. *Nucleic Acids Res.* 41 D590–D596. 10.1093/nar/gks1219 23193283 PMC3531112

[B72] Raskin L. Stromley J. M. Rittmann B. E. Stahl D. A. (1994). Group-specific 16S rRNA hybridization probes to describe natural communities of methanogens. *Appl. Environ. Microbiol.* 60 1232–1240. 7517128 10.1128/aem.60.4.1232-1240.1994 PMC201464

[B73] Reed H. E. Martiny J. B. (2013). Microbial composition affects the functioning of estuarine sediments. *ISME J.* 7 868–879. 10.1038/ismej.2012.154 23235294 PMC3603390

[B74] Rojo F. (2009). Degradation of alkanes by bacteria. *Environ. Microbiol.* 11 2477–2490. 10.1111/j.1462-2920.2009.01948.x 19807712

[B75] Rosano-Hernández M. C. Ramírez-Saad H. Fernández-Linares L. (2012). Petroleum-influenced beach sediments of the campeche bank, Mexico: diversity and bacterial community structure assessment. *J. Environ. Manage.* 95 S325–S331. 10.1016/j.jenvman.2011.06.046 21802196

[B76] Sauret C. Séverin T. Vétion G. Guigue C. Goutx M. Pujo-Pay M. (2014). “Rare biosphere” bacteria as key phenanthrene degraders in coastal seawaters. *Environ. Pollut.* 194 246–253. 10.1016/j.envpol.2014.07.024 25156140

[B77] Sauret C. Tedetti M. Guigue C. Dumas C. Lami R. Pujo-Pay M. (2015). Influence of PAHs among other coastal environmental variables on total and PAH-degrading bacterial communities. *Environ. Sci. Pollut. Res. Int.* 23 4242–4256. 10.1007/s11356-015-4768-0 26122564

[B78] Schloss P. D. Westcott S. L. Ryabin T. Hall J. R. Hartmann M. Hollister E. B. (2009). Introducing mothur: open-source, platform-independent, community-supported software for describing and comparing microbial communities. *Appl. Environ. Microbiol.* 75 7537–7541. 10.1128/AEM.01541-09 19801464 PMC2786419

[B79] Solé R. V. Montoya J. M. (2001). Complexity and fragility in ecological networks. *Proc. R. Soc. B Biol. Sci.* 268 2039–2045. 10.1098/rspb.2001.1767 PMC1088846 11571051

[B80] Sridhar H. Srinivasan U. Askins R. A. Canales-Delgadillo J. C. Chen C.-C. Ewert D. N. (2012). Positive relationships between association strength and phenotypic similarity characterize the assembly of mixed-species bird flocks worldwide. *Am. Nat.* 180 777–790. 10.1086/668012 23149402

[B81] Stauffert M. Cravo-Laureau C. Duran R. (2014a). Dynamic of sulphate-reducing microorganisms in petroleum-contaminated marine sediments inhabited by the polychaete hediste diversicolor. *Environ. Sci. Pollut. Res.* 22 15273–15284. 10.1007/s11356-014-3624-y 25256587

[B82] Stauffert M. Cravo-Laureau C. Jézéquel R. Barantal S. Cuny P. Gilbert F. (2013). Impact of oil on bacterial community structure in bioturbated sediments. *PLoS ONE* 8:e65347 10.1371/journal.pone.0065347 PMC3677869 23762350

[B83] Stauffert M. Duran R. Gassie C. Cravo-Laureau C. (2014b). Response of archaeal communities to oil spill in bioturbated mudflat sediments. *Microb. Ecol.* 67 108–119. 10.1007/s00248-013-0288-y 24057322

[B84] Steele J. A. Countway P. D. Xia L. Vigil P. D. Beman J. M. Kim D. Y. (2011). Marine bacterial, archaeal and protistan association networks reveal ecological linkages. *ISME J.* 5 1414–1425. 10.1038/ismej.2011.24 21430787 PMC3160682

[B85] Stone L. Roberts A. (1990). The checkerboard score and species distributions. *Oecologia* 85 74–79. 10.1007/BF00317345 28310957

[B86] Suárez-Suárez A. López-López A. Tovar-Sánchez A. Yarza P. Orfila A. Terrados J. (2011). Response of sulfate-reducing bacteria to an artificial oil-spill in a coastal marine sediment. *Environ. Microbiol.* 13 1488–1499. 10.1111/j.1462-2920.2011.02451.x 21414123

[B87] Sun M. Y. Dafforn K. A. Brown M. V. Johnston E. L. (2012). Bacterial communities are sensitive indicators of contaminant stress. *Mar. Pollut. Bull.* 64 1029–1038. 10.1016/j.marpolbul.2012.01.035 22385752

[B88] Sun M. Y. Dafforn K. A. Johnston E. L. Brown M. V. (2013). Core sediment bacteria drive community response to anthropogenic contamination over multiple environmental gradients. *Environ. Microbiol.* 15 2517–2531. 10.1111/1462-2920.12133 23647974

[B89] Teira E. Lekunberri I. Gasol J. M. Nieto-Cid M. Alvarez-Salgado X. A. Figueiras F. G. (2007). Dynamics of the hydrocarbon-degrading *Cycloclasticus* bacteria during mesocosm-simulated oil spills. *Environ. Microbiol.* 9 2551–2562. 10.1111/j.1462-2920.2007.01373.x 17803779

[B90] Tronczynski J. Musnchy C. Héas-Moisan K. Guiot N. Truquet I. (2005). Analyse de contaminants organiques (PCB, OCP, HAP) dans les sédiments marins. Ifremer: Méthodes d’analyses en milieu marin, 1637–1844.

[B91] Ulrich W. Ollik M. (2004). Frequent and occasional species and the shape of relative-abundance distributions. *Divers. Distrib.* 10 263–269. 10.1111/j.1366-9516.2004.00082.x

[B92] Ulrich W. Zalewski M. (2006). Abundance and co-occurrence patterns of core and satellite species of ground beetles on small lake islands. *Oikos* 114 338–348. 10.1111/j.2006.0030-1299.14773.x

[B93] Vick-Majors T. J. Priscu J. C. Amaral-Zettler L. A. (2014). Modular community structure suggests metabolic plasticity during the transition to polar night in ice-covered antarctic lakes. *ISME J.* 8 778–789. 10.1038/ismej.2013.190 24152712 PMC3960534

[B94] Weisburg W. G. Barns S. M. Pelletier D. A. Lane D. J. (1991). 16S ribosomal DNA amplification for phylogenetic study. *J. Bacteriol.* 173 697–703. 1987160 10.1128/jb.173.2.697-703.1991 PMC207061

[B95] Wiatrowski H. A. Barkay T. (2005). Monitoring of microbial metal transformations in the environment. *Curr. Opin. Biotechnol.* 16 261–268. 10.1016/j.copbio.2005.04.011 15961026

[B96] Zhang W. Ki J.-S. Qian P.-Y. (2008). Microbial diversity in polluted harbor sediments i: bacterial community assessment based on four clone libraries of 16S rDNA. *Estuar. Coast. Shelf Sci.* 76 668–681. 10.1016/j.ecss.2007.07.040

[B97] Zhou J. Deng Y. Luo F. He Z. Tu Q. Zhi X. (2010). Functional molecular ecological networks. *mBio* 1 e169–e110. 10.1128/mBio.00169-10 PMC2953006 20941329

[B98] Zhou J. Deng Y. Luo F. He Z. Yang Y. (2011). phylogenetic molecular ecological network of soil microbial communities in response to elevated CO2. *mBio* 2 e122–e111. 10.1128/mBio.00122-11 PMC3143843 21791581

