# Supplementary material for: Response of Core Microbial Consortia to Chronic Hydrocarbon Contaminations in Coastal Sediment Habitats
Source: Front Microbiol. 2016 Oct 13;7:1637. doi: 10.3389/fmicb.2016.01637 (PMC5061854; doi:10.3389/fmicb.2016.01637)
Supplement: Supplementary file 3 [file Data_Sheet_3.docx]

Supplementary Material

Response of core microbial consortia to hydrocarbon contaminations in coastal sediment habitats

Mathilde Jeanbille*, Jérôme Gury, Robert Duran, Jacek Tronczynski, Jean-François Ghiglione, Hélène Agogué, Olfa ben Saïd and Jean-Christophe Auguet

*** Correspondence:** Corresponding Author: mathilde.jeanbille@univ-pau.fr

# Supplementary table

**Supplementary table 1 :** Geographical description, environmental parameters, PAH concentrations (ppb) and ratios. ND = Not Determined.

|  |  |  |  |  |  |  |  |  |  |
| --- | --- | --- | --- | --- | --- | --- | --- | --- | --- |
|  | **Geographic areas** | **Longitudes** | **Latitudes** | **Month and year of sampling** | **Water column depth (m)** | **Water column salinity (g/l)** | **Water column temperature (°C)** | **Granulometry (%>63µm)** | **% TOC** |
| **BA1** | **Vermilion Coast** | 3.135056 | 42.490889 | 04-2012 | 6 | 37.8 | 16.4 | 43.3 | 6.1 |
| **BA2** |  | 3.135056 | 42.490889 | 07-2012 | 6 | 37.8 | 16.4 | 6.7 | 1.7 |
| **BA3** |  | 3.135056 | 42.490889 | 11-2012 | 6 | 37.8 | 16.4 | 24.3 | 4.5 |
| **BA4** |  | 3.135056 | 42.490889 | 02-2013 | 6 | 37.8 | 16.4 | 20.7 | 3.7 |
| **BA5** |  | 3.149167 | 42.491917 | 04-2012 | 24 | 37.8 | 16.4 | 5.8 | 1.5 |
| **PV1** |  | 3.120583 | 42.521639 | 04-2012 | 14 | 37.8 | 16.4 | 52.1 | 7.0 |
| **PV2** |  | 3.120583 | 42.521639 | 07-2012 | 14 | 37.8 | 16.4 | 57.1 | 6.0 |
| **PV3** |  | 3.120583 | 42.521639 | 02-2013 | 24 | 37.8 | 16.4 | 11.2 | 2.9 |
| **PV4** |  | 3.125278 | 42.530583 | 11-2012 | 14 | 37.8 | 16.4 | 59.3 | 6.3 |
| **PV5** |  | 3.125278 | 42.530583 | 04-2012 | 24 | 37.8 | 16.4 | 3.8 | 2.1 |
| **BI1** | **Bizerte** | 9.926008 | 37.186503 | 04-2012 | 2 | 29 | 23.7 | ND | 5.1 |
| **BI2** |  | 9.838863 | 37.141826 | 04-2012 | 2 | 28.8 | 23.2 | ND | 4.1 |
| **BI3** |  | 9.785814 | 37.187591 | 04-2012 | 2 | 27 | 23.1 | ND | 2.1 |
| **BI4** |  | 9.873962 | 37.266868 | 04-2012 | 2 | 32.2 | 23.4 | ND | 3.5 |
| **L1** | **Lebannon** | 33.916163 | 35.544857 | 07-2007 | ND | 39.3 | 22.5 | ND | ND |
| **L2** |  | 33.916163 | 35.544857 | 07-2007 | ND | 39.3 | 22.5 | ND | ND |
| **L3** |  | 33.263700 | 35.082110 | 07-2007 | 300 | 39.3 | 22.5 | 100.0 | 0.9 |
| **M3** | **Gulf of Lion** | 5.909040 | 43.083000 | 02-2004 | 8.5 | 37.9 | 17 | 61.8 | ND |
| **M4** |  | 5.909040 | 43.083000 | 07-2010 | 8 | 37.9 | 17 | 78.9 | 6.0 |
| **M5** |  | 3.380500 | 43.242900 | 05-2004 | 12 | 37.5 | 16.5 | 78.5 | 4.2 |
| **M6** |  | 3.571333 | 43.452800 | 04-2006 | 2.2 | 37.5 | 16.5 | 90.8 | 3.6 |
| **M11** |  | 4.402219 | 43.404000 | 08-2005 | 20 | 35.5 | 17.7 | 75.0 | 1.4 |
| **M12** |  | 4.402219 | 43.404000 | 08-2010 | 17 | 35.5 | 17.7 | 77.7 | 0.9 |
| **M13** |  | 3.571333 | 43.452800 | 05-2004 | 6 | 37.5 | 16.5 | 23.0 | 6.7 |
| **M1** | **Corsica** | 8.754998 | 41.920000 | 03-2004 | 65 | 38.1 | 18.2 | 67.8 | ND |
| **M2** |  | 8.754998 | 41.920000 | 07-2010 | 37 | 38.1 | 18.2 | 7.9 | 2.2 |
| **M7** |  | 8.670991 | 42.277000 | 03-2004 | 87 | 38.1 | 18.2 | 68.9 | ND |
| **M8** |  | 8.670991 | 42.277000 | 07-2010 | 91 | 38.1 | 18.2 | 75.5 | 4.1 |
| **M9** |  | 9.299997 | 42.731000 | 03-2004 | 99 | 38.1 | 13.5 | 39.4 | ND |
| **M10** |  | 9.299997 | 42.731000 | 07-2010 | 96 | 38.1 | 13.5 | 36.5 | 1.3 |
| **GC1** | **Bay of Biscay** | -3.074845 | 47.496700 | 06-1999 | 12.2 | 34.8 | 13.5 | 33.4 | 0.8 |
| **GC2** |  | -3.428251 | 47.666700 | 06-1999 | 27.6 | 34.8 | 13.5 | 46.9 | 3.1 |
| **GC3** |  | -3.365356 | 47.703700 | 06-1999 | 20.2 | 34.8 | 13.5 | 20.5 | 0.9 |
| **GC4** |  | -2.791339 | 47.454000 | 06-1999 | 74.4 | 34.4 | 13 | 55.6 | 1.0 |
| **GC5** |  | -2.791339 | 47.454000 | 06-1999 | 14.2 | 34.4 | 13 | 40.5 | 1.1 |
| **GC6** |  | -2.015060 | 47.010600 | 07-2000 | 0 | 33.7 | 13.5 | 51.2 | 2.6 |
| **GC7** |  | -2.015100 | 47.010600 | 08-1999 | 0 | 33.7 | 13.5 | 38.1 | 1.9 |
| **MA1** | **English channel** | -0.276001 | 49.505300 | 06-2009 | 16 | 34.8 | 11.7 | 8.9 | 0.9 |
| **MA2** |  | -1.638377 | 49.672100 | 06-2009 | 15 | 34.8 | 11.7 | 5.6 | 0.6 |
| **MA3** |  | -1.776359 | 48.949000 | 07-2001 | 12.3 | 35 | 12.3 | 26.7 | 2.7 |
| **LR1** | **La Rochelle** | -1.166972 | 46.145660 | 10-2013 | 2 | 33.6 | 19 | ND | ND |
| **LR2** |  | -1.162000 | 46.122000 | 10-2013 | ND | 33.6 | 19 | ND | ND |

**Supplementary table 1** (continued)

|  |  |  | **Bulk PAHs concentrations (ppb)** | | | | | | | | | | |
| --- | --- | --- | --- | --- | --- | --- | --- | --- | --- | --- | --- | --- | --- |
|  | **Phenanthrene/Anthracene** | **Fluoranthene/Pyrene** | **Fluoranthene** | **Pyrene** | **Anthracene** | **Benz[*a*]anthracene** | **Benzo[*a*]pyrene** | **Benzo[*ghi*]perylene** | **Chrysene** | **Dibenz[*a,h*]anthracene** | **Fluorene** | **Indeno[1,2,3-*cd*]Pyrene** | **Phenanthrene** |
| **BA1** | 2.76 | 1.33 | 231 | 173 | 59 | 208 | 285 | 107 | 106 | 33 | 39 | 236 | 162 |
| **BA2** | 5.18 | 1.38 | 96 | 70 | 11 | 28 | 29 | 24 | 22 | 4 | 6 | 29 | 57 |
| **BA3** | 2.91 | 0.70 | 56 | 79 | 20 | 85 | 91 | 36 | 36 | 11 | 13 | 74 | 58 |
| **BA4** | 3.14 | 0.86 | 471 | 545 | 144 | 496 | 421 | 165 | 169 | 44 | 79 | 307 | 453 |
| **BA5** | 4.68 | 1.28 | 14 | 11 | 3 | 9 | 17 | 11 | 7 | 2 | 1 | 18 | 12 |
| **PV1** | 2.06 | 1.19 | 869 | 728 | 161 | 1072 | 1318 | 443 | 471 | 151 | 42 | 976 | 332 |
| **PV2** | 2.47 | 1.32 | 119 | 91 | 24 | 106 | 116 | 57 | 40 | 19 | 9 | 122 | 59 |
| **PV3** | 2.76 | 1.26 | 793 | 630 | 106 | 700 | 954 | 362 | 251 | 96 | 41 | 703 | 294 |
| **PV4** | 4.58 | 1.45 | 99 | 69 | 1 | 70 | 74 | 33 | 29 | 10 | 15 | 69 | 5 |
| **PV5** | 3.36 | 1.23 | 20 | 16 | 2 | 17 | 12 | 12 | 10 | 4 | 1 | 28 | 7 |
| **BI1** | 9.44 | 0.58 | 8 | 13 | 3 | 2 | 3 | 8 | 2 | 5 | 4 | 4 | 24 |
| **BI2** | 4.80 | 0.74 | 66 | 90 | 25 | 31 | 42 | 78 | 22 | 19 | 56 | 74 | 118 |
| **BI3** | 3.28 | 1.24 | 110 | 89 | 25 | 60 | 51 | 64 | 52 | 31 | 30 | 162 | 81 |
| **BI4** | 8.98 | 0.64 | 2 | 4 | 1 | 1253 | 969 | 734 | 582 | 166 | 24 | 644 | 5 |
| **L1** | 8.39 | 0.46 | 20 | 42 | 6 | 22 | 13 | 30 | 43 | 7 | 39 | 25 | 47 |
| **L2** | 4.47 | 0.57 | 16 | 29 | 5 | 16 | 10 | 22 | 22 | 13 | 15 | 19 | 20 |
| **L3** | 7.78 | 1.07 | 6 | 6 | 1 | 3 | 3 | 4 | 4 | 1 | 1 | 5 | 6 |
| **M3** | 2.97 | 0.81 | 1198 | 1482 | 217 | 1065 | 1293 | 775 | 1304 | 234 | 32 | 849 | 645 |
| **M4** | 4.60 | 1.22 | 1540 | 1260 | 111 | 870 | 990 | 716 | 934 | 160 | 1 | 822 | 511 |
| **M5** | 3.96 | 1.12 | 1042 | 933 | 124 | 544 | 696 | 549 | 674 | 140 | 51 | 565 | 493 |
| **M6** | 3.97 | 1.34 | 1640 | 1220 | 83 | 672 | 694 | 545 | 666 | 144 | 18 | 726 | 331 |
| **M11** | 13.33 | 1.20 | 12 | 10 | 1 | 6 | 4 | 5 | 12 | 5 | 4 | 5 | 16 |
| **M12** | 14.30 | 1.32 | 15 | 11 | 1 | 5 | 4 | 9 | 9 | 5 | 1 | 6 | 14 |
| **M13** | 5.83 | 1.12 | 57 | 51 | 6 | 32 | 37 | 33 | 36 | 9 | 4 | 38 | 34 |
| **M1** | 1.44 | 0.93 | 1300 | 1405 | 369 | 933 | 1305 | 1165 | 1255 | 298 | 25 | 1195 | 531 |
| **M2** | 3.16 | 1.11 | 1680 | 1520 | 164 | 767 | 771 | 589 | 858 | 102 | 31 | 632 | 518 |
| **M7** | 5.86 | 1.09 | 60 | 55 | 3 | 32 | 30 | 31 | 38 | 6 | 1 | 30 | 17 |
| **M8** | 12.40 | 1.08 | 55 | 51 | 1 | 26 | 29 | 24 | 31 | 5 | 1 | 26 | 12 |
| **M9** | 4.50 | 1.02 | 5 | 5 | 1 | 3 | 3 | 5 | 3 | 5 | 1 | 5 | 5 |
| **M10** | 1.00 | 1.22 | 2 | 2 | 1 | 2 | 1 | 5 | 3 | 5 | 1 | 5 | 1 |
| **GC1** | 5.10 | 0.97 | 547 | 564 | 68 | 306 | 260 | 182 | 317 | 34 | 18 | 217 | 347 |
| **GC2** | 8.75 | 1.03 | 448 | 434 | 71 | 176 | 157 | 116 | 216 | 21 | 140 | 138 | 621 |
| **GC3** | 13.69 | 0.99 | 373 | 376 | 13 | 192 | 112 | 123 | 239 | 27 | 13 | 147 | 178 |
| **GC4** | 5.91 | 1.15 | 23 | 20 | 2 | 16 | 10 | 9 | 21 | 5 | 2 | 12 | 13 |
| **GC5** | 4.44 | 1.13 | 26 | 23 | 3 | 17 | 19 | 22 | 22 | 5 | 2 | 34 | 12 |
| **GC6** | 3.75 | 1.01 | 67 | 66 | 8 | 40 | 52 | 47 | 31 | 13 | 4 | 49 | 31 |
| **GC7** | 5.77 | 1.35 | 36 | 26 | 3 | 14 | 19 | 20 | 34 | 4 | 3 | 25 | 18 |
| **MA1** | 5.32 | 1.25 | 819 | 654 | 145 | 440 | 361 | 319 | 338 | 55 | 46 | 513 | 772 |
| **MA2** | 4.00 | 1.31 | 323 | 247 | 43 | 151 | 139 | 97 | 156 | 15 | 5 | 169 | 173 |
| **MA3** | 9.00 | 1.67 | 10 | 6 | 1 | 3 | 4 | 5 | 6 | 5 | 2 | 9 | 9 |
| **LR1** | 11.77 | 1.04 | 352 | 338 | 51 | 136 | 288 | 207 | 270 | 63 | 406 | 287 | 598 |
| **LR2** | 6.49 | 1.03 | 175 | 170 | 6 | 76 | 67 | 59 | 89 | 42 | 17 | 51 | 41 |

**Supplementary table 2:** Pyrosequencing dataset description**.**

|  |  | **Bacteria** | **Archaea** | **Eukarya** |
| --- | --- | --- | --- | --- |
| **raw reads** | mean nb. / sample | 15228 | 4822 | 2139 |
|  | total | 639687 | 202519 | 110044 |
| **quality-checked reads** | mean nb. / sample | 9336 | 2730 | 1964 |
|  | total | 392398 | 114654 | 82468 |
|  | mean length (pb) | 273 | 287 | 158 |

**Supplementary table 3:** Environmental variables used in analysis, according to Spearman correlation (p<0.05). Correlations are based on 32 samples.

| Variables used in analysis | Correlated variables \|R^2^\|>0.75 |
| --- | --- |
| **Salinity** | Salinity |
|  | Temperature |
| **Latitude** | Latitude |
| **PSD** | PSD |
| **%TOC** | %TOC |
| **Fluoranthene/Pyrene** | Fluoranthene/Pyrene |
| **Phenanthrene/Anthracene** | Phenanthrene/Anthracene |
| **Fluoranthene** | Fluoranthene, Pyrene, Anthracene, Benz[*a*]anthracene, Benzo[*a*]pyrene, Benzo[*g,h,i*]Perylene, Chrysene, Dibenz[*a,h*]Anthracene, Indeno[1,2,3,*c-d*]Pyrene, Phenanthrene |

# Supplementary Figures

**Supplementary Figure 1:** Hierarchical classification analysis of euclidean distances between the 42 samples based on PAH concentrations for the Mediterranean Sea (A) and the Atlantic (B). Clusters of contaminated (black) and non-contaminated (grey) samples are indicated for each region.

**Supplementary figure 2:** Comparisons between Mediterranean and Atlantic sites of means of environmental parameters**.** P-values were calculated using the Wilcoxon-Mann-Whitney U test or the Student’s t-Test after testing the homoscedasticity of the data.

**Supplementary Figure S3:** Plot of the phenanthrene to anthracene (P/A) ratio against the fluoranthene to pyrene (F/P) ratio. Each dot stands for one sediment sample. P/A values above 10 and F/P values below 1 indicate petrogenic input of hydrocarbons. Conversly, P/A values below 10 and F/P values above 1 indicate pyrogenic input of hydrocarbons.
